# *N*-(4-chloro­benzo­yl)-*N*-(2-chloro­phen­yl)-*O*-[2-(2-nitro­phen­yl)acet­yl]hydroxyl­amine

**DOI:** 10.1107/S1600536812040883

**Published:** 2012-10-03

**Authors:** Jing Ma, Yi Ma, Dian He

**Affiliations:** a Institute of Medicinal Chemistry, School of Pharmacy, Lanzhou University, Lanzhou 730000, Gansu Province, People’s Republic of China; b Gansu College of Traditional Chinese Medicine, Lanzhou 730000, Gansu Province, People’s Republic of China

## Abstract

In the title hydroxamic acid derivate, C_21_H_14_N_2_O_5_Cl_2_, the nitro-substituted benzene ring forms dihedral angles of 66.0 (2) and 59.6 (2)°, with the *p*-chloro and *o*-chloro-substituted benzene rings, respectively. The dihedral angle between the two chloro-substituted benzene rings is 64.2 (2) Å. In the crystal, weak C—H⋯O hydrogen bonds link the mol­ecules along [010]. The crystal studied was an inversion twin with refined components in the ratio 0.60 (7):0.40 (7).

## Related literature

For applications of hydroxamic acid derivatives, see: Noh *et al.* (2009 ▶); Zeng *et al.* (2003 ▶). For the synthesis, see: Ayyangark *et al.* (1986 ▶). For a related structure, see: Zhang *et al.* (2012 ▶).
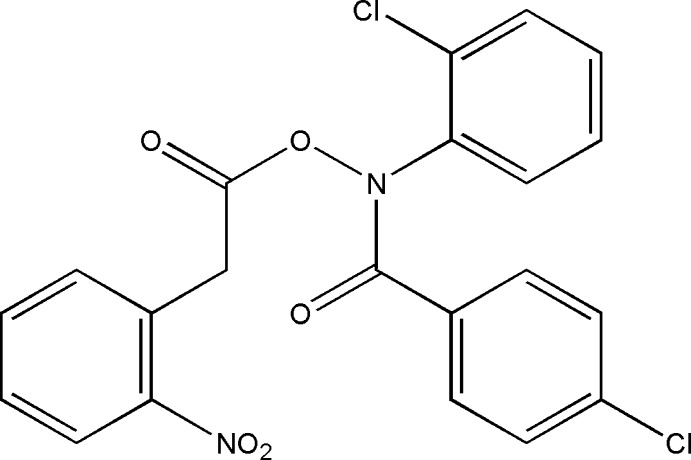


## Experimental

### 

#### Crystal data

C_21_H_14_Cl_2_N_2_O_5_
*M* *_r_* = 445.24
Monoclinic, 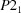

*a* = 12.366 (14) Å
*b* = 6.789 (8) Å
*c* = 12.579 (14) Å
β = 105.150 (14)°
*V* = 1019 (2) Å^3^
*Z* = 2
Mo *K*α radiation
μ = 0.36 mm^−1^
*T* = 296 K
0.21 × 0.20 × 0.16 mm

#### Data collection

Bruker APEXII CCD diffractometer
Absorption correction: multi-scan (*SADABS*; Sheldrick, 1996 ▶) *T*_min_ = 0.929, *T*_max_ = 0.945
5091 measured reflections
3669 independent reflections
1821 reflections with *I* > 2σ(*I*)
*R*_int_ = 0.030

#### Refinement

*R*[*F* ^2^ > 2σ(*F* ^2^)] = 0.054
*wR*(*F* ^2^) = 0.069
*S* = 0.99
3669 reflections
271 parameters
1 restraint
H-atom parameters constrained
Δρ_max_ = 0.17 e Å^−3^
Δρ_min_ = −0.16 e Å^−3^
Absolute structure: Flack (1983 ▶), 1624 Friedel pairs
Flack parameter: 0.40 (7)

### 

Data collection: *APEX2* (Bruker, 2009 ▶); cell refinement: *SAINT* (Bruker, 2009 ▶); data reduction: *SAINT*; program(s) used to solve structure: *SHELXS97* (Sheldrick, 2008 ▶); program(s) used to refine structure: *SHELXL97* (Sheldrick, 2008 ▶); molecular graphics: *PLATON* (Spek, 2009 ▶); software used to prepare material for publication: *SHELXL97*.

## Supplementary Material

Crystal structure: contains datablock(s) global, I. DOI: 10.1107/S1600536812040883/lh5536sup1.cif

Structure factors: contains datablock(s) I. DOI: 10.1107/S1600536812040883/lh5536Isup2.hkl

Additional supplementary materials:  crystallographic information; 3D view; checkCIF report

## Figures and Tables

**Table 1:** Hydrogen-bond geometry (Å, °)

*D*—H⋯*A*	*D*—H	H⋯*A*	*D*⋯*A*	*D*—H⋯*A*
C5—H5⋯O1^i^	0.93	2.38	3.264 (7)	158
C15—H15*B*⋯O1^ii^	0.97	2.45	3.421 (6)	175

Symmetry codes: (i) 
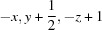
; (ii) 
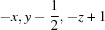
.

**Fig. 1.**
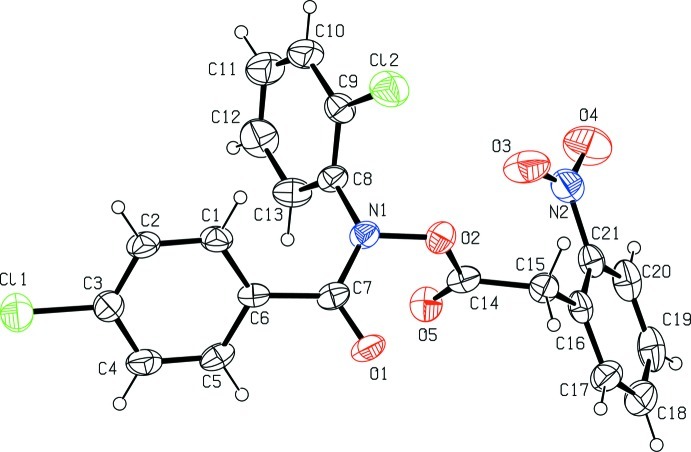
The molecular structure of the title compound with 30% probability displacement ellipsoids. H atoms are shown as small spheres of arbitrary radius.

**Fig. 2.**
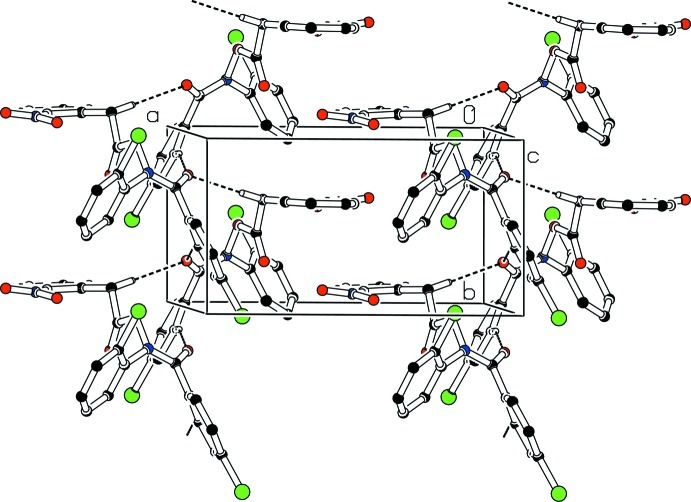
Part of the crystal structure with weak hydrogen bonds shown as dashed lines.

## supplementary crystallographic information

### Comment

Hydroxamic acid derivatives have received considerable attention in recent years as the result of the discovery of their role in the biochemical toxicology of many drugs and other chemicals (Noh *et al.*, 2009; Zeng *et al.*, 2003). The molecular structure of the title compound is sjown in Fig. 1. The nitro-substituted benzene ring (C16-C24) forms dihedral angles of 66.0 (2) and 59.6 (2)°, with the 4-chloro (C1-C6) and 2-chloro-substituted (C8-C13) benzene rings, respectively. The dihedral angle between the two chloro-substituted benzene rings (C1-6/C8-C13) is 64.2 (2)Å. In the crystal, weak C—H···O hydrogen bonds linke molecules along [010] (Fig .2). The bond legths and angles can be compared to those in N-(2-Chlorophenyl)-1-phenylformamido 3-(2-nitrophenyl)propanoate (Zhang *et al.*, 2012).

### Experimental

The title compound (I) was prepared according to the method described by Ayyangark *et al.* (1986). Crystals suitable for single-crystal X-ray analysis were grown by slow evaporation of a solution of (I) in dichloromethane-methanol (1:3 *v*/*v*).

### Refinement

Hydrogen atoms were placed in calculated positions with C—H = 0.93 and 0.97Å and included in a riding-model approximation with *U*_iso_ = 1.2*U*_eq_(C).

### Crystal data

**Table:**

C_21_H_14_Cl_2_N_2_O_5_	*F*(000) = 456
*M**_r_* = 445.24	*D*_x_ = 1.450 Mg m^−^^3^
Monoclinic, *P*2_1_	Mo *K*α radiation, λ = 0.71073 Å
Hall symbol: P 2yb	Cell parameters from 985 reflections
*a* = 12.366 (14) Å	θ = 2.7–19.2°
*b* = 6.789 (8) Å	µ = 0.36 mm^−^^1^
*c* = 12.579 (14) Å	*T* = 296 K
β = 105.150 (14)°	Block, colorless
*V* = 1019 (2) Å^3^	0.21 × 0.20 × 0.16 mm
*Z* = 2	

### Data collection

**Table:**

Bruker APEXII CCD diffractometer	3669 independent reflections
Radiation source: fine-focus sealed tube	1821 reflections with *I* > 2σ(*I*)
Graphite monochromator	*R*_int_ = 0.030
φ and ω scans	θ_max_ = 25.5°, θ_min_ = 2.7°
Absorption correction: multi-scan (*SADABS*; Sheldrick, 1996)	*h* = −14→14
*T*_min_ = 0.929, *T*_max_ = 0.945	*k* = −8→8
5091 measured reflections	*l* = −15→14

### Refinement

**Table:**

Refinement on *F*^2^	H-atom parameters constrained
Least-squares matrix: full	*w* = 1/[σ^2^(*F*_o_^2^) + (0.0117*P*)^2^] where *P* = (*F*_o_^2^ + 2*F*_c_^2^)/3
*R*[*F*^2^ > 2σ(*F*^2^)] = 0.054	(Δ/σ)_max_ < 0.001
*wR*(*F*^2^) = 0.069	Δρ_max_ = 0.17 e Å^−^^3^
*S* = 0.99	Δρ_min_ = −0.16 e Å^−^^3^
3669 reflections	Absolute structure: Flack (1983), 1624 Friedel pairs
271 parameters	Flack parameter: 0.40 (7)
1 restraint	

### Special details

**Table:**

Geometry. All e.s.d.'s (except the e.s.d. in the dihedral angle between two l.s. planes) are estimated using the full covariance matrix. The cell e.s.d.'s are taken into account individually in the estimation of e.s.d.'s in distances, angles and torsion angles; correlations between e.s.d.'s in cell parameters are only used when they are defined by crystal symmetry. An approximate (isotropic) treatment of cell e.s.d.'s is used for estimating e.s.d.'s involving l.s. planes.
Refinement. Refinement of *F*^2^ against ALL reflections. The weighted *R*-factor *wR* and goodness of fit *S* are based on *F*^2^, conventional *R*-factors *R* are based on *F*, with *F* set to zero for negative *F*^2^. The threshold expression of *F*^2^ > σ(*F*^2^) is used only for calculating *R*-factors(gt) *etc*. and is not relevant to the choice of reflections for refinement. *R*-factors based on *F*^2^ are statistically about twice as large as those based on *F*, and *R*- factors based on ALL data will be even larger.

### Fractional atomic coordinates and isotropic or equivalent isotropic displacement parameters (Å^2^)

**Table:**

	*x*	*y*	*z*	*U*_iso_*/*U*_eq_	
C1	0.0147 (3)	0.5015 (8)	0.8371 (3)	0.0556 (12)	
H1	0.0422	0.4007	0.8869	0.067*	
C2	−0.0321 (4)	0.6675 (7)	0.8709 (3)	0.0590 (13)	
H2	−0.0363	0.6786	0.9434	0.071*	
C3	−0.0720 (4)	0.8152 (6)	0.7973 (4)	0.0581 (14)	
C4	−0.0663 (4)	0.8041 (7)	0.6904 (4)	0.0642 (15)	
H4	−0.0935	0.9061	0.6414	0.077*	
C5	−0.0194 (4)	0.6382 (7)	0.6562 (4)	0.0554 (13)	
H5	−0.0148	0.6292	0.5838	0.067*	
C6	0.0207 (3)	0.4855 (7)	0.7292 (3)	0.0439 (11)	
C7	0.0602 (4)	0.3045 (7)	0.6835 (4)	0.0493 (13)	
C8	0.2401 (3)	0.3019 (7)	0.8323 (4)	0.0487 (12)	
C9	0.2722 (4)	0.2095 (7)	0.9344 (4)	0.0577 (13)	
C10	0.3508 (4)	0.2985 (10)	1.0199 (5)	0.0814 (17)	
H10	0.3720	0.2376	1.0884	0.098*	
C11	0.3970 (4)	0.4729 (11)	1.0044 (5)	0.0906 (19)	
H11	0.4484	0.5328	1.0628	0.109*	
C12	0.3684 (4)	0.5626 (8)	0.9024 (5)	0.0831 (18)	
H12	0.4026	0.6801	0.8915	0.100*	
C13	0.2899 (4)	0.4789 (8)	0.8174 (4)	0.0640 (14)	
H13	0.2697	0.5411	0.7492	0.077*	
C14	0.2324 (3)	0.0798 (8)	0.6138 (4)	0.0493 (13)	
C15	0.2469 (3)	−0.1148 (6)	0.5610 (3)	0.0523 (12)	
H15A	0.2688	−0.2138	0.6182	0.063*	
H15B	0.1754	−0.1543	0.5128	0.063*	
C16	0.3330 (4)	−0.1087 (6)	0.4954 (4)	0.0494 (12)	
C17	0.2956 (4)	−0.1148 (7)	0.3812 (4)	0.0669 (14)	
H17	0.2190	−0.1217	0.3485	0.080*	
C18	0.3679 (5)	−0.1107 (8)	0.3152 (4)	0.0791 (16)	
H18	0.3406	−0.1116	0.2390	0.095*	
C19	0.4823 (5)	−0.1054 (7)	0.3634 (5)	0.0778 (16)	
H19	0.5320	−0.1053	0.3191	0.093*	
C20	0.5227 (4)	−0.1003 (7)	0.4756 (5)	0.0690 (14)	
H20	0.5994	−0.0966	0.5082	0.083*	
C21	0.4474 (4)	−0.1006 (6)	0.5391 (4)	0.0511 (12)	
Cl1	−0.13093 (10)	1.0228 (2)	0.84135 (10)	0.0924 (4)	
Cl2	0.21125 (11)	−0.0100 (2)	0.95615 (10)	0.0868 (4)	
N1	0.1548 (3)	0.2169 (6)	0.7465 (3)	0.0501 (9)	
N2	0.4985 (4)	−0.0959 (6)	0.6589 (4)	0.0711 (13)	
O1	0.0103 (2)	0.2380 (4)	0.5951 (2)	0.0622 (9)	
O2	0.1866 (2)	0.0409 (4)	0.7008 (2)	0.0563 (8)	
O3	0.4389 (3)	−0.0664 (6)	0.7204 (3)	0.0983 (14)	
O4	0.5971 (3)	−0.1181 (7)	0.6925 (3)	0.1191 (16)	
O5	0.2537 (2)	0.2392 (5)	0.5864 (2)	0.0613 (10)	

### Atomic displacement parameters (Å^2^)

**Table:**

	*U*^11^	*U*^22^	*U*^33^	*U*^12^	*U*^13^	*U*^23^
C1	0.062 (3)	0.066 (3)	0.040 (3)	0.008 (3)	0.015 (2)	0.012 (3)
C2	0.068 (3)	0.077 (4)	0.035 (3)	0.006 (3)	0.017 (3)	0.005 (3)
C3	0.053 (3)	0.053 (3)	0.062 (4)	−0.007 (3)	0.005 (3)	−0.006 (3)
C4	0.068 (4)	0.065 (4)	0.048 (4)	−0.012 (3)	−0.006 (3)	0.005 (3)
C5	0.063 (4)	0.070 (4)	0.035 (3)	−0.020 (3)	0.016 (3)	−0.002 (3)
C6	0.038 (3)	0.058 (3)	0.035 (3)	−0.005 (3)	0.007 (2)	0.007 (3)
C7	0.050 (3)	0.068 (4)	0.036 (3)	−0.006 (3)	0.022 (3)	0.003 (3)
C8	0.029 (3)	0.071 (4)	0.043 (3)	0.003 (3)	0.005 (3)	−0.008 (3)
C9	0.046 (3)	0.065 (3)	0.061 (3)	0.011 (3)	0.010 (3)	0.001 (3)
C10	0.067 (4)	0.103 (5)	0.061 (4)	0.024 (4)	−0.007 (3)	−0.005 (4)
C11	0.076 (4)	0.108 (6)	0.074 (4)	0.011 (5)	−0.006 (3)	−0.012 (5)
C12	0.055 (4)	0.083 (5)	0.104 (5)	−0.008 (4)	0.008 (3)	−0.004 (4)
C13	0.049 (3)	0.082 (4)	0.057 (3)	−0.009 (3)	0.007 (3)	0.012 (3)
C14	0.031 (3)	0.076 (4)	0.042 (3)	−0.004 (3)	0.011 (2)	0.000 (3)
C15	0.042 (3)	0.059 (3)	0.055 (3)	−0.001 (3)	0.010 (2)	−0.006 (3)
C16	0.040 (3)	0.042 (3)	0.068 (4)	0.007 (3)	0.017 (3)	−0.004 (3)
C17	0.067 (4)	0.082 (3)	0.051 (3)	0.009 (3)	0.013 (3)	−0.011 (3)
C18	0.101 (5)	0.082 (4)	0.063 (4)	0.014 (4)	0.036 (4)	−0.003 (3)
C19	0.083 (5)	0.055 (3)	0.114 (5)	0.009 (4)	0.059 (4)	−0.005 (4)
C20	0.050 (3)	0.050 (3)	0.110 (5)	−0.003 (3)	0.025 (3)	−0.012 (4)
C21	0.049 (3)	0.038 (3)	0.069 (4)	−0.001 (3)	0.019 (3)	0.000 (3)
Cl1	0.0909 (10)	0.0798 (10)	0.0966 (10)	0.0165 (10)	0.0068 (8)	−0.0179 (9)
Cl2	0.1014 (10)	0.0849 (11)	0.0788 (9)	0.0064 (10)	0.0320 (7)	0.0200 (9)
N1	0.040 (2)	0.065 (3)	0.044 (2)	0.003 (2)	0.009 (2)	−0.006 (2)
N2	0.060 (4)	0.059 (3)	0.083 (4)	−0.002 (3)	−0.002 (3)	−0.004 (3)
O1	0.057 (2)	0.088 (2)	0.0363 (19)	0.001 (2)	0.0039 (16)	−0.0121 (18)
O2	0.0591 (19)	0.060 (2)	0.0549 (19)	−0.0036 (19)	0.0230 (16)	−0.0048 (18)
O3	0.073 (3)	0.146 (4)	0.067 (3)	0.002 (3)	0.003 (2)	0.003 (3)
O4	0.056 (2)	0.155 (4)	0.124 (3)	0.012 (3)	−0.016 (2)	−0.003 (3)
O5	0.063 (2)	0.062 (2)	0.067 (2)	−0.006 (2)	0.0312 (18)	0.0019 (18)

### Geometric parameters (Å, º)

**Table:**

C1—C6	1.382 (5)	C12—C13	1.366 (5)
C1—C2	1.384 (6)	C12—H12	0.9300
C1—H1	0.9300	C13—H13	0.9300
C2—C3	1.366 (6)	C14—O5	1.186 (5)
C2—H2	0.9300	C14—O2	1.384 (5)
C3—C4	1.367 (5)	C14—C15	1.510 (6)
C3—Cl1	1.742 (5)	C15—C16	1.509 (5)
C4—C5	1.386 (5)	C15—H15A	0.9700
C4—H4	0.9300	C15—H15B	0.9700
C5—C6	1.388 (5)	C16—C21	1.379 (5)
C5—H5	0.9300	C16—C17	1.390 (5)
C6—C7	1.492 (6)	C17—C18	1.370 (6)
C7—O1	1.209 (5)	C17—H17	0.9300
C7—N1	1.367 (5)	C18—C19	1.386 (6)
C8—C13	1.385 (5)	C18—H18	0.9300
C8—C9	1.391 (5)	C19—C20	1.369 (6)
C8—N1	1.419 (5)	C19—H19	0.9300
C9—C10	1.386 (6)	C20—C21	1.378 (5)
C9—Cl2	1.724 (5)	C20—H20	0.9300
C10—C11	1.351 (6)	C21—N2	1.474 (6)
C10—H10	0.9300	N1—O2	1.426 (4)
C11—C12	1.381 (6)	N2—O4	1.191 (5)
C11—H11	0.9300	N2—O3	1.216 (4)
			
C6—C1—C2	120.0 (4)	C12—C13—H13	119.8
C6—C1—H1	120.0	C8—C13—H13	119.8
C2—C1—H1	120.0	O5—C14—O2	124.9 (5)
C3—C2—C1	119.8 (4)	O5—C14—C15	127.6 (4)
C3—C2—H2	120.1	O2—C14—C15	107.5 (4)
C1—C2—H2	120.1	C16—C15—C14	113.5 (4)
C2—C3—C4	121.5 (4)	C16—C15—H15A	108.9
C2—C3—Cl1	119.1 (4)	C14—C15—H15A	108.9
C4—C3—Cl1	119.4 (4)	C16—C15—H15B	108.9
C3—C4—C5	118.9 (4)	C14—C15—H15B	108.9
C3—C4—H4	120.5	H15A—C15—H15B	107.7
C5—C4—H4	120.5	C21—C16—C17	116.3 (4)
C4—C5—C6	120.5 (4)	C21—C16—C15	125.5 (4)
C4—C5—H5	119.7	C17—C16—C15	118.3 (4)
C6—C5—H5	119.7	C18—C17—C16	122.2 (5)
C1—C6—C5	119.3 (5)	C18—C17—H17	118.9
C1—C6—C7	123.4 (5)	C16—C17—H17	118.9
C5—C6—C7	117.2 (4)	C17—C18—C19	119.3 (5)
O1—C7—N1	121.7 (4)	C17—C18—H18	120.4
O1—C7—C6	121.5 (4)	C19—C18—H18	120.4
N1—C7—C6	116.8 (4)	C20—C19—C18	120.5 (5)
C13—C8—C9	119.1 (4)	C20—C19—H19	119.8
C13—C8—N1	121.2 (4)	C18—C19—H19	119.8
C9—C8—N1	119.6 (5)	C19—C20—C21	118.6 (5)
C10—C9—C8	119.5 (5)	C19—C20—H20	120.7
C10—C9—Cl2	120.1 (5)	C21—C20—H20	120.7
C8—C9—Cl2	120.4 (4)	C20—C21—C16	123.2 (5)
C11—C10—C9	120.5 (6)	C20—C21—N2	114.8 (5)
C11—C10—H10	119.7	C16—C21—N2	122.0 (4)
C9—C10—H10	119.7	C7—N1—C8	128.2 (4)
C10—C11—C12	120.3 (6)	C7—N1—O2	114.5 (3)
C10—C11—H11	119.8	C8—N1—O2	114.8 (3)
C12—C11—H11	119.8	O4—N2—O3	122.0 (5)
C13—C12—C11	120.1 (6)	O4—N2—C21	119.0 (5)
C13—C12—H12	120.0	O3—N2—C21	118.9 (4)
C11—C12—H12	120.0	C14—O2—N1	111.9 (3)
C12—C13—C8	120.4 (4)		
			
C6—C1—C2—C3	−0.1 (7)	C14—C15—C16—C17	−107.7 (5)
C1—C2—C3—C4	−0.5 (7)	C21—C16—C17—C18	−0.7 (7)
C1—C2—C3—Cl1	179.9 (3)	C15—C16—C17—C18	−179.7 (4)
C2—C3—C4—C5	0.4 (7)	C16—C17—C18—C19	1.6 (8)
Cl1—C3—C4—C5	−180.0 (3)	C17—C18—C19—C20	−1.3 (8)
C3—C4—C5—C6	0.3 (7)	C18—C19—C20—C21	0.0 (8)
C2—C1—C6—C5	0.7 (6)	C19—C20—C21—C16	1.0 (7)
C2—C1—C6—C7	−174.2 (4)	C19—C20—C21—N2	179.7 (4)
C4—C5—C6—C1	−0.8 (6)	C17—C16—C21—C20	−0.6 (7)
C4—C5—C6—C7	174.4 (4)	C15—C16—C21—C20	178.3 (4)
C1—C6—C7—O1	134.5 (4)	C17—C16—C21—N2	−179.3 (4)
C5—C6—C7—O1	−40.6 (6)	C15—C16—C21—N2	−0.3 (7)
C1—C6—C7—N1	−45.1 (5)	O1—C7—N1—C8	159.2 (4)
C5—C6—C7—N1	139.9 (4)	C6—C7—N1—C8	−21.3 (6)
C13—C8—C9—C10	1.7 (6)	O1—C7—N1—O2	−1.6 (5)
N1—C8—C9—C10	−176.3 (4)	C6—C7—N1—O2	177.9 (3)
C13—C8—C9—Cl2	179.5 (3)	C13—C8—N1—C7	−49.9 (6)
N1—C8—C9—Cl2	1.5 (6)	C9—C8—N1—C7	128.1 (4)
C8—C9—C10—C11	−0.5 (7)	C13—C8—N1—O2	110.9 (4)
Cl2—C9—C10—C11	−178.4 (4)	C9—C8—N1—O2	−71.2 (5)
C9—C10—C11—C12	−1.5 (8)	C20—C21—N2—O4	−9.7 (7)
C10—C11—C12—C13	2.5 (8)	C16—C21—N2—O4	169.1 (5)
C11—C12—C13—C8	−1.3 (7)	C20—C21—N2—O3	169.2 (4)
C9—C8—C13—C12	−0.8 (6)	C16—C21—N2—O3	−12.1 (7)
N1—C8—C13—C12	177.2 (4)	O5—C14—O2—N1	7.0 (6)
O5—C14—C15—C16	23.1 (6)	C15—C14—O2—N1	−171.7 (3)
O2—C14—C15—C16	−158.2 (3)	C7—N1—O2—C14	74.1 (4)
C14—C15—C16—C21	73.3 (6)	C8—N1—O2—C14	−89.4 (4)

### Hydrogen-bond geometry (Å, º)

**Table:**

*D*—H···*A*	*D*—H	H···*A*	*D*···*A*	*D*—H···*A*
C5—H5···O1^i^	0.93	2.38	3.264 (7)	158
C15—H15*B*···O1^ii^	0.97	2.45	3.421 (6)	175

Symmetry codes: (i) −*x*, *y*+1/2, −*z*+1; (ii) −*x*, *y*−1/2, −*z*+1.
